# Online cognitive behavioral therapy for insomnia (CBT-I) for the treatment of insomnia among individuals with alcohol use disorder: study protocol for a randomized controlled trial

**DOI:** 10.1186/s40814-018-0376-3

**Published:** 2018-12-10

**Authors:** Alyssa T. Brooks, Ralph T. Tuason, Subhajit Chakravorty, Shravya Raju, Lee M. Ritterband, Frances P. Thorndike, Gwenyth R. Wallen

**Affiliations:** 1 National Institutes of Health Clinical Center, Bethesda, USA; 2 Perelman School of Medicine, Department of Psychiatry, University of Pennsylvania, Philadelphia, USA; 3 Department of Psychiatry and Neurobehavioral Sciences, Center for Behavioral Health and Technology, PO Box 801075, Charlottesville, VA 22908 USA; 4 Pear Therapeutics, Boston, USA

**Keywords:** Alcohol use disorder, Insomnia, Cognitive behavioral therapy for insomnia, Sleep disturbance, Internet intervention

## Abstract

**Abstract:**

Alcohol use disorder (AUD) is characterized by problematic drinking that becomes severe. Individuals with AUD often experience insomnia and other sleep disturbances at various phases of recovery. Cognitive behavioral therapy for insomnia (CBT-I) is an efficacious non-pharmacological treatment for insomnia and is recommended as a first-line treatment for adults with chronic insomnia. Internet-based CBT-I could play a key role in the dissemination of this behavioral sleep intervention, given the paucity of trained clinicians able to provide CBT-I in person and other logistical/cost concerns. SHUTi (Sleep Healthy Using The Internet) is the most tested and empirically-sound Internet intervention for insomnia. Despite the promise of Internet-based CBT-I interventions, to date, no randomized controlled trials (RCTs) exist examining the feasibility/efficacy of an Internet-based CBT-I program among treatment-seeking individuals recovering from AUD. This is a two-phase RCT assessing feasibility/acceptability and efficacy of the SHUTi program among individuals with AUD in recovery with insomnia. Phase I will focus on assessing the feasibility and acceptability of program delivery and data collection (*n* = 10). Phase II will be an RCT powered to examine preliminary intervention efficacy (*n* = 30 per group). Participants for this study must meet criteria for “moderate to severe” insomnia. Individuals randomized to the intervention group will receive the SHUTi intervention (initiated while inpatient and completed while outpatient), and individuals randomized to the control group will receive an educational web-based program. The goals of the study are as follows: (1) assess the feasibility and acceptability of Internet-based CBT-I among individuals with AUD in recovery with insomnia (phase I), (2) compare the preliminary efficacy of CBT-I versus control group with respect to primary and secondary outcome variables (phase II), and (3) explore specific domains associated with improved outcomes, e.g., demographic, psychiatric, and drinking-related factors (phase II). Primary outcome measures include changes in insomnia severity over time and changes in actigraphy-recorded sleep efficiency over time.

**Trial registration:**

NCT#03493958; registered 1 June 2018.

**Electronic supplementary material:**

The online version of this article (10.1186/s40814-018-0376-3) contains supplementary material, which is available to authorized users.

## Background

### Alcohol and sleep disturbances

Alcohol use disorder (AUD) is characterized by problematic drinking that becomes severe [1]. To be diagnosed with “severe” AUD, individuals must meet six or more of the 11 criteria outlined in the Diagnostic and Statistical Manual (current edition: DSM-5). This was referred to as “alcohol dependence” in previous versions of the DSM; thus, some researchers and clinicians still use the term “dependence” and research conducted prior to the release of the DSM-5 uses the term “dependence.” Alcohol dependence is associated with insomnia and a myriad of other sleep-related disorders [2–4]. Insomnia in individuals who are alcohol-dependent and actively drinking may aggravate existing psychosocial problems [5]. The prevalence of insomnia in individuals with alcohol dependence is estimated to be between 36 and 91%. After 2 weeks of alcohol detoxification, as many as 65% of these individuals still experience sleep problems [2].

### Cognitive behavioral therapy for insomnia (CBT-I) as a first-line treatment for insomnia

CBT-I is an efficacious non-pharmacological treatment for insomnia [6] and is recommended as a first-line treatment for adults with chronic insomnia disorder [7]. It is also effective for those with posttraumatic stress disorder (PTSD) and depression, which are common co-morbidities among those with AUD [8, 9]. CBT-I has been associated with more rapid and durable improvement in sleep outcomes, even when compared with other non-pharmacologic treatments [10, 11]. While pharmacological intervention for insomnia combined with CBT-I can produce added benefits acutely, medications should ultimately be discontinued for long-term outcomes during “maintenance” CBT-I in order to reduce the chance of insomnia remission [12].

### Efficacy of Internet-delivered CBT-I

Internet-based CBT-I could play a key role in the dissemination of behavioral sleep interventions, given the paucity of trained clinicians able to provide CBT-I in-person and other logistical/cost concerns. This method of delivery is of particular interest to researchers and clinicians working with individuals with AUD [13]. A recent systematic review and meta-analysis of 11 randomized controlled trials (total of 1460 participants) revealed that online CBT-I improved insomnia severity, sleep efficiency, subjective sleep quality, wake after sleep onset, sleep onset latency, total sleep time, and number of nocturnal awakenings with effect sizes comparable to face-to-face CBT-I [14]. Another meta-analysis showed that Internet-based CBT-I significantly improved comorbid anxiety and depression [15]. The most recent meta-analysis of 15 studies showed improved sleep efficiency and total sleep time, while decreasing insomnia severity and depressive symptoms that was maintained from 4 to 48 weeks after the post-treatment assessment [16].

### Description of Sleep Health Using the Internet (SHUTi) program

**Fig. 1:**
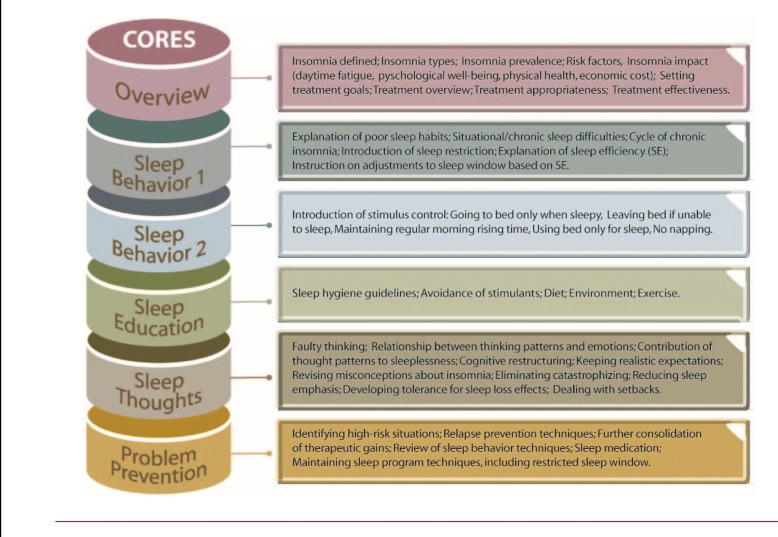
SHUTi Cores and core descriptions. Adapted from “Development and Perceived Utility and Impact of an Internet Intervention for Insomnia,” by Thorndike et al. [21]

The SHUTi program is an automated, interactive, Internet-based intervention based on well-established face-to-face CBT-I components including sleep restriction, stimulus control, sleep hygiene, cognitive restructuring, and relapse prevention. It consists of six core areas of focus (Fig. 1) that include interactive educational content and case studies about insomnia and its precipitating factors. These Cores are designed to parallel traditional (in-person) CBT-I sessions and are “metered” out over time; that is, new Cores are available seven days after completion of the previous core. The program includes a variety of interactive features including goal-setting, feedback based on user-entered data and user-identified symptoms, animations, quizzes, vignettes, and video-based expert explanations. SHUTi was developed based on the Model for Internet Interventions [17, 41]. Sleep diary input is used to customize the program to individual situations. In adults with primary insomnia, SHUTi significantly improved the Insomnia Severity Index (ISI) scores and these improvements were sustained at a 6-month follow-up. Additionally, there were significant decreases in wake after sleep onset (WASO) and increases in sleep efficiency compared to a wait-list control group [17, 41], with similar effects in adult cancer survivors [18]. Evaluation of co-occurring symptoms in those trials showed that SHUTi also significantly improved psychological symptoms, mental health-related quality of life, and fatigue [19]. More recently, researchers established long-term (1 year follow-up) effectiveness in a representative sample with chronic insomnia and a range of comorbid conditions via RCT [20]. Finally, the program has significantly lowered depressive symptoms among those with both insomnia and depression symptoms (without major depressive disorder) compared to time- and attention-matched controls [22].

### Gap in the literature/purpose of study

Despite the established efficacy of CBT-I in the community and in those with AUD, very little is known about the efficacy of Internet-based CBT-I among individuals with AUD [13]. In phase I of the trial described herein, we will assess the feasibility and acceptability of Internet-based CBT-I among treatment-seeking individuals with AUD in recovery. In phase II, we will compare the efficacy of CBT-I versus an education-only control group with respect to primary and secondary outcome variables. Primary outcome variables include changes in both self-reported insomnia severity and actigraphy-recorded sleep efficiency over time. Secondary outcome variables include additional actigraphy-related outcomes, alcohol craving and consumption, sleep disturbance, daytime sleepiness, anxiety/depression, fatigue, self-efficacy for sleep, dysfunctional beliefs about sleep, and functional outcomes.

## Study design and methods

All participants enrolled in this study will have first been admitted to a clinical research facility providing rehabilitation treatment (NIH Clinical Center) under a screening and assessment protocol, which enrolls adults over 18 years of age seeking inpatient treatment for AUD. Once consented and enrolled into inpatient treatment, screening, and research protocol, participants are screened and recruited to participate in additional studies for which they meet eligibility criteria (including the study described herein). In phase I of this study, all participants will be given access to the SHUTi intervention. Phase I will assess the feasibility and acceptability of the intervention and ensure that our data collection processes are functioning as they should be. Participants will receive training from a member of the research team on accessing the program and using the handheld tablets provided as part of study participation.

In phase II, we will randomize participants to each of the two conditions. The study team will remain blind to the randomization scheme until each study participant is deemed eligible and signs the consent form. Permuted block randomization with a block size of four will be used to equally assign participants to each group. The statistician, who will not have any contact with participants, will prepare the randomization sequences and provide the allocation assignment after each participant is consented. Individuals assigned to the intervention group will complete six sessions (“Cores”) of SHUTi intervention (at least one Core completed while inpatient, the rest while outpatient). The SHUTi program tailors specific recommendations based on sleep diary responses or other input within the program (e.g., responses on the Dysfunctional Beliefs and Attitude Scale trigger recommendations for specific cognitive restructuring strategies). The SHUTi program has the ability to send automated pre-developed introductory, reminder, congratulatory, and (insomnia) relapse prevention emails to participants, which serve to enhance engagement and clinical outcomes. Individuals assigned to the control group will have access to an insomnia education web-based program that participants access and read at their own pace. The program contains information about insomnia symptoms, impacts, and causes, when to see a doctor, and basic strategies to improve sleep. While the content of the educational program overlaps with that of SHUTi, the education-only program contains no interactive features and delivers content all at once (participants need not wait for content to be “unlocked” over time). Additionally, the educational program contains no customization of the program based on sleep diary responses. Instead, participants in the control group are given educational information (like they might see on WebMD or National Sleep Foundation websites), but are left to apply it themselves.

Study participants will be asked to come back to the NIH Clinical Center post-discharge for a follow-up visit to complete a face-to-face semi-structured interview (phase I participants only) and assessments (phases I and II participants). If participants are unable to return for the follow-up visit, we will attempt to conduct the surveys and interview via phone. Participants who are randomly assigned to the intervention condition will be granted access to the program free of charge for up to 6 months from the start of the program. Participants who are randomly assigned to the education-only condition will be given access to the full SHUTi program 3 months after discharge upon request. Similarly, participants who are randomly assigned to the intervention condition will be granted access to the educational materials provided to the education-only condition participants 3 months after discharge upon request.

## Study measures

### Screening measures

Screening measures will include the Insomnia Severity Index (ISI), which is a seven-item global index of self-reported insomnia symptom severity [23] and an objective measure of each individual’s Apnea-Hypopnea Index (AHI) to determine presence/severity of obstructive sleep apnea (OSA) measured with the WatchPAT. The WatchPAT is a portable diagnostic medical device that works by using Peripheral Arterial Tone (PAT) technology that allows for non-invasive and portable detection of sleep apnea [24]. WatchPAT-generated AHI was shown to have a high correlation with polysomnography (PSG)-generated AHI (*r* = 0.88–0.92) making it a good alternative to PSG as an OSA diagnostic tool [25–27]. The results of the WatchPAT study will be reviewed with the patient and the clinical care team. If the patient’s results suggest *mild* obstructive sleep apnea (5–14.9 events/hour of sleep), we will recommend him/her to work on losing weight (if they are overweight or obese), avoid alcohol use, and follow up with a primary care provider. In the case that they are found to have *moderate to severe* OSA (score of 15 or higher), we will provide the patient with a patient education handout that explains what OSA is, the risks associated with untreated OSA, and the treatment options available. We will urge the patient to seek evaluation and treatment through a primary care provider or directly from a sleep medicine clinic, as is appropriate for them.

### Feasibility and acceptability measures

Participants enrolled during phase I will be asked to start the SHUTi Cores prior to being discharged from inpatient treatment. They will be invited to participate in a short interview after they complete the first SHUTi Core in order to gather additional information on program delivery and acceptability. We will also utilize the Internet Evaluation and Utility Questionnaire [28] at the pre-discharge interview, which is a 16-item measure designed to assess usability, likeability, usefulness, understandability, and convenience of the intervention. Open-ended questions (used in phase I only) will be audio recorded and the entire session (including the questionnaires) is expected to last between 20 and 60 min, depending on participants’ responses.

We will implement an exit assessment for phase I *and* II participants. At the exit assessment, we will use the Internet Impact and Effectiveness Questionnaire, the Internet Evaluation and Utility Questionnaire, and the Internet Intervention Adherence Questionnaire [28]. Participants will complete these assessments with a member of the research team who will take notes on participants’ comments for each question. Additional measures of program adherence (in the experimental group, for both phases I and II) will be measured by three variables: login count (goal of two per week), completed diary count (minimum of five per week), and number of “Cores” completed out of a possible six Cores.

### Outcome measures

Outcome measures administered at the pre- and post-test assessment (see Additional file 1 for timing of administration) include the Insomnia Severity Index (ISI), daily sleep and symptom diaries, the Penn Alcohol Craving Scale [29], the Functional Outcomes of Sleep Questionnaire (FOSQ-10; [30]), the Pittsburgh Sleep Quality Index (PSQI; [31]), the Multidimensional Fatigue Symptom Inventory-Short Form (MFSI-SF; [32]), the Epworth Sleepiness Scale (ESS; [33]), the Self-Efficacy for Sleep Scale (SE-S; [34]), the Dysfunctional Beliefs and Attitudes about Sleep Scale (brief version; DBAS-16; [35]), the Inventory of Depressive Symptoms (IDS; 30-item self-rated version; [36]), the Trait Anxiety Inventory [37], the Composite Scale of Morningness (CSM; [38]), and measures of relapse to include a dichotomous (yes/no) assessment and Timeline Follow-Back (TLFB; [39]).

### Objective measure of sleep: actigraphy

“Actiwatches” are small wristband data loggers which contain accelerometers and light sensors in order to objectively assess sleep. We have used the *Actiwatch Spectrum Plus* (Philips Respironics) in previous studies with patients undergoing alcohol rehabilitation in both inpatient [40] and outpatient settings. All study participants will be asked to wear an actiwatch for 4 days (capturing three nights’ worth of sleep data) at three study time points. The main outcome of interest will be improvements in sleep efficiency from baseline to follow-up assessment periods. The watch also provides measures of total sleep time, wake after sleep onset, sleep onset latency, number of awakenings, and time in bed.

### Inclusion and exclusion criteria

Participants will be eligible for this study if they are at least 18 years of age, self-report “moderate or severe” insomnia as indicated by a score of 15 or higher on the Insomnia Severity Index, are admitted as a treatment-seeking inpatient at the NIH Clinical Center under protocol 14-AA-0181 (signing both the clinical and research consent), have been an inpatient for at least 14 days prior to consent/screening, can speak, understand, and write in English, and are able to comply with study requirements (including ability to access the Internet at least two times per week). Participants will be ineligible for this study if they are over the age of 65, report a physician diagnosis of moderate to severe obstructive sleep apnea (OSA) or test positive for moderate to severe OSA as documented with an Apnea Hypopnea Index of ≥ 15 events/hour based on WatchPAT testing results, have irregular sleep schedules that prevent the ability to follow treatment recommendations (i.e., usual bedtimes outside of 8:00 pm to 2:00 am or arising times outside of 4:00 am to 10:00 am), meet criteria for severe opioid and/or cocaine use disorder in the past year, meet criteria for moderate to severe cannabis use disorder in the past year, meet diagnostic criteria for an unstable or serious psychiatric condition (schizophrenia, bipolar, major depressive disorder not currently in remission), based on the SCID for DSM-5, or have an unstable or serious medical/neurologic illness as identified by the Principal Investigator or Medical Advisory Investigator. Participants will be discontinued/withdrawn from the study if any of the following events occur: participant requests to be removed from study, participant is admitted to an addiction inpatient treatment program for 2 weeks or longer, participant exhibits any condition which the Medical Advisory Investigator finds CBT-I to be a hazard to the participant (i.e., participant seems agitated, onset of increasing paranoia), participant is unable to comply with study-related procedures (i.e., SHUTi program recommendations and/or “homework”), and/or participant develops a medical illness or condition that requires a new hospital admission.

### Power analysis

In studies of CBT-I, the effect sizes for improvements in insomnia severity ranged from *d* = 0.2 [20] to *d* = 2.24 [18], with the sample sizes ranging from 29 [18] to 300 individuals [20]. One such study also provided effect sizes for improvements in several other sleep-related variables, including wake after sleep onset (*d* = 0.74), sleep onset latency (*d* = 0.26), and sleep efficiency (*d* = 0.68; [17, 41]). In phase I, our sample size is based on the concept of *saturation*, which describes the number of participants at which no new (qualitative) key themes are likely to be generated. Based on our previous qualitative research in the same population, it is expected that 12 patients will be screened to achieve 10 “completers” (i.e., individuals providing pre- and post-test assessment data). The total sample size for phase II of this study will test the primary hypotheses associated with the analysis plan. With insomnia severity as the primary outcome, we will use a two-sided alternative hypothesis that the rate of change will be different between the intervention group and the control group. Assuming a correlation of 0.6 between the two time points, an alpha level of 0.05, a power level of 0.8, and an estimated attrition rate of 40%, we will be able to detect an effect size of 0.84 with 60 participants [42].

### Justification for specific inclusion/exclusion criteria

Clinical insomnia is considered to be moderate to severe insomnia and is the target of treatment (refer to Bastien et al. 2001 [23], Fig. 1 for reference). In order to demonstrate efficacy, treatment in patients with “clinical” insomnia is considered appropriate and will help obtain estimates of effect sizes. Sub-clinical insomnia may remit on its own without treatment, thus making it unclear whether the intervention improved insomnia or it was a case of spontaneous remission of insomnia. One of CBT-I’s potential side effects from its sleep restriction phase is increased daytime sleepiness. Increased daytime sleepiness is associated with increased frequency of falls in the elderly (individuals over 65 years old; [43]); thus, we are excluding individuals over the age of 65. Among individuals who are alcohol-dependent with untreated moderate-severe OSA, sleep restriction may further increase sympathetic and stress response, leading to an increased risk of cardiovascular adverse events [44]. Severe OSA is associated with increased daytime sleepiness, motor vehicle crashes, depression, cardiovascular and cerebral morbidity and mortality, and cognitive and metabolic dysfunction [45]. Thus, we are excluding individuals with untreated moderate to severe OSA. A study by Pacek et al. [46] reported that participants reported sleep difficulties with abstinence from cannabis and resulted in the relapse in cannabis use at a later time. It is unclear whether sleep difficulty symptoms improve with relapse, but the authors also point out that cannabis use contributes to observed sleep difficulties. Whether or not disordered sleep contributes to long-term cannabis use is unclear [46]. Finally, cannabis is independently associated with impairment of sleep continuity [46]. Opioids are associated with a more complex picture of sleep disorders that includes insomnia, obstructive sleep apnea, and complex sleep apnea [47]. Thus, we are excluding participants with severe opioid/cocaine use disorder in the past year and/or moderate-severe cannabis use disorder in the past year. After enrollment in the study, the management of sedatives/hypnotics for individuals who have been prescribed either will be done by a clinician who is blind to the treatment condition.

### Recruitment plan

All eligible participants admitted to the NIH Clinical Center alcohol rehabilitation unit who signed the clinical and research consents for the screening/treatment protocol will be approached for participation in this study. The Principal Investigator or a trained Associate Investigator approved by the IRB will explain the study objectives, time commitment, expectations, and processes for assessments.

## Statistical analysis

### Qualitative analysis (phase I only)

Each audio-recorded interview will be transcribed verbatim. First, independent coders will review all transcripts and identify common themes. Once the theme list is agreed on, after thematic analysis, a codebook will be developed based on themes from the interviews. Each code will be accompanied by an operational definition that will allow for clarity and consistency in the coding process. Evidence of each code or theme will be assessed using quotes from the interviews. A team of coders will independently review all transcripts. Discordant coding will be discussed until consensus among the coding team is achieved. Once the iterative process of consensus building is complete, an intramural expert in qualitative methodology will validate the final themes and coding. If this final reviewer notes any discrepancies during this validation process or if they identify an additional theme not previously noted, the coded themes will be returned to the team of coders for further consensus. After data are coded, NVivo will be utilized for further qualitative analysis and data management.

### Quantitative analyses (phase II only)

Patterns of missing data will be examined thoroughly to assess whether any questions were systematically skipped by all participants or any sub-group of participants. Initial analyses will be descriptive and exploratory in nature to identify any changes in insomnia severity (as measured by the ISI) over time. Mixed models for repeated measure with group, time, and the interaction of group by time will be used to test the changes of insomnia severity in two groups. Mixed models for repeated measures will also be used to test the secondary objectives with potential covariates in the model. Demographic, psychiatric, and/or drinking-related factors significantly related to the outcomes listed in the secondary objectives will be entered in the model. Akaike information criterion and the Bayesian information criterion will be used to select best-fitting models. A potential dosing effect analysis including the number of Cores completed as a covariate will be conducted for the treatment group only. With the linear mixed model approach, all available data points will be included in the analyses. Under missing completely at random and missing at random assumptions, a linear mixed model with restricted maximum likelihood estimation can produce unbiased estimators [48].

### Actigraphy analyses

After device removal and data download, raw data from the *Actiwatch Spectrums* will be analyzed using the Philips Respironics computerized sleep scoring software, which scores each epoch based on a threshold method algorithm. Investigators will review each sleep period prior to analysis to screen for malfunctioning watches, corrupt data, and required adjustments using bedtimes and wake times from self-reported diary data when necessary.

### Evaluation of risks/discomforts and benefits ratio

This study will provide pilot data on the feasibility, acceptability, and preliminary efficacy of an Internet-based CBT-I program undergoing inpatient treatment AUD. The program may represent an efficacious non-pharmacologic intervention for insomnia in this population, potentially decreasing the need and use of a pharmacological intervention. However, it is possible that there may be no direct benefit to the participant. It is possible that answering some of the questionnaires may cause discomfort to participants. Participants may feel uncomfortable wearing the WatchPAT device or the actiwatch device to sleep. Participants may experience a rash and/or pruritus while wearing the WatchPAT or actiwatch. Participants in phase I and those assigned to the SHUTi condition in phase II may experience daytime sleepiness (and potentially impaired daytime functioning), particularly if they are taking medications with side effects of drowsiness during the study period. Every effort will be made to address and minimize participant discomfort including scheduling a repeat session or postponing study procedures if a participant becomes uncomfortable. Confidentiality and information technology security standards are in place as part of the NIH Clinical Center intramural program to protect electronic repositories of patient data. We selected a minimally invasive diagnostic tool to assess for OSA, requiring only one night of sleeping with the device. It is reasonably expected that these safeguards will protect participants’ medical and personal health information, ensuring their privacy.

### Data/records management

The PI will be responsible for assuring that all investigators follow the plan for protecting the confidentiality of information and data provided by research participants. Information to be collected via the Internet-based insomnia treatment program, SHUTi, will include participants’ responses to individual questions within the program and the dates and times questions and Cores are completed. Participants will not be asked to provide any personally identifiable information in the course of their engagement in the SHUTi intervention and/or education-only programs. At the initial meeting for consenting and baseline assessment, participants will be provided with a unique user login ID and a temporary password. The participant will be required to change the password at initial logon to the website to a personal one that he or she will recall. Only the participant and the study staff will have the authority to access the participant’s responses to the components of the intervention. All baseline and follow-up assessments not part of the SHUTi program will be completed in person or over the phone with paper-and-pencil questionnaires and will be administered by a member of the research team.

### Compensation

Participants will be compensated for research-related discomfort and inconveniences in accordance with NIH guidelines. If participants are unable to finish the study, they will be paid only for those parts completed. Because participants will need access to a tablet or laptop to complete the program, we will provide tablets to all participants (capable of Wifi connectivity). Participants will be able to keep the study-provided tablets.

### Strengths and limitations

This study is not without possible limitations. Participants must be comfortable using a tablet; thus, study team members will provide informal training. Also, all participants will be recruited from a clinical research facility providing rehabilitation and must be able to comply with study requirements (i.e., using a tablet) so it may be difficult to generalize results. For instance, participants spend an average of about 31 days in inpatient treatment, and sleep hygiene on the inpatient unit may be more regulated compared to at home. Cross-contamination of study arms may occur, particularly since participants will be in close proximity in an inpatient setting. Participants will be encouraged to not share information about SHUTi or the education component with other patients on the unit. The strengths of this study lie in the use of both subjective and objective (actigraphy) assessments of sleep, validated patient-reported outcome measures, and high potential for dissemination in real-world settings. Examining potential correlates of intervention outcome will allow us to identify further sub-groups of individuals to target for interventions in the future.

## Conclusions

This study addresses a critical gap in our understanding of the utility of Internet-based CBT-I in a vulnerable population. While insomnia and other sleep disturbances are prevalent among individuals with AUD, to date, no randomized controlled trials have been conducted to establish the feasibility, acceptability, and preliminary efficacy of an Internet-based CBT-I program among individuals with comorbid insomnia and AUD. Understanding the utility of SHUTi and other behavioral interventions like it could help clinicians who support individuals with AUD in recovery identify useful, accessible, and efficacious treatments for use in this population. From a public health perspective, a low-cost, low-risk behavioral intervention for insomnia such as SHUTi could be implemented widely in both clinical and community settings.

## Trial status

This study was approved by the NIH Addictions Institutional Review Board in March of 2018 (NCT#03493958). We expect to begin recruitment in June of 2018.

## Additional file

Additional file 1: Study Schema and Timing of Assessments. Timing of assessment of outcome measures. (DOCX 19 kb)

## Abbreviations

AUD: Alcohol use disorder
CBT-I: Cognitive behavioral therapy for insomnia
CSM: Composite Scale of Morningness
DSM: Diagnostic and Statistical Manual
ESS: Epworth Sleepiness Scale
FOSQ: Functional Outcomes of Sleep Questionnaire
ISI: Insomnia Severity Index
MFSI-SF: Multidimensional Fatigue Symptom Inventory-Short Form
NIH: National Institutes of Health
PACS: Penn Alcohol Craving Scale
PSQI: Pittsburgh Sleep Quality Index
SE-S: Self-Efficacy for Sleep Scale
SHUTi: Sleep Healthy Using the Internet
