# Supplementary material for: Online cognitive behavioral therapy for insomnia (CBT-I) for the treatment of insomnia among individuals with alcohol use disorder: study protocol for a randomized controlled trial
Source: Pilot Feasibility Stud. 2018 Dec 10;4:183. doi: 10.1186/s40814-018-0376-3 (PMC6287341; doi:10.1186/s40814-018-0376-3)
Supplement: Supplementary file 1 — Study Schema and Timing of Assessments. Timing of assessment of outcome measures. (DOCX 19 kb) [file 40814_2018_376_MOESM1_ESM.docx]

|  | **STUDY PERIOD** | | | | | | | | |
| --- | --- | --- | --- | --- | --- | --- | --- | --- | --- |
|  | **Inpatient** | | | | | | **Outpatient** | | |
|  | **Screening** | **Enrollment** | **Randomization** | **Pre-Web program* initiation** | **Start Web program** | **Pre-discharge** | **SHUTi/Insomnia Education Program** | **SHUTi/Insomnia Education Program Completion** | **Long-Term Follow-up** |
| **TIMELINE** | **Day 14** | **Day 15-16** | **Day 17** | **Day 18** | **Day 22** | **Day 22 – Discharge** | **3-4 weeks post web program initiation** | **9-11 weeks post web program initiation** | **6-7 months post discharge** |
| **Informed Consent** |  | X |  |  |  |  |  |  |  |
| **OSA Testing** |  | X |  |  |  |  |  |  |  |
| **SHUTi (Intervention Group)** |  |  | X |  | X | Complete 1^st^ core |  |  |  |
|  |  |  | Or |  | Or |  |  |  |  |
| **Insomnia Education Web-Based Program**  **(Control group)** |  |  | X |  | X |  |  |  |  |
| **Study Measures:** | | | | | | | | | |
| **ISI** | X |  |  |  |  |  | X |  | X |
| **MFSI-SF** |  |  |  | X |  |  |  | X |  |
| **PSQI** |  |  |  | X |  |  | X | X |  |
| **DBAS-16** |  |  |  | X |  |  |  | X |  |
| **FOSQ-10** |  |  |  | X |  |  |  | X |  |
| **SE-S** |  |  |  | X |  |  |  | X |  |
| **IDS** |  |  |  | X |  |  |  | X |  |
| **ESS** |  |  |  | X |  |  |  | X |  |
| **TAI** |  |  |  | X |  |  |  | X |  |
| **CSM** |  |  |  | X |  |  |  | X |  |
| **PACS** |  |  |  |  |  |  |  | X | X |
| **Actigraphy (3-4 days)** |  |  |  | X |  |  | X | X |  |
| **Sleep Diaries** |  |  |  | X  (Online) |  |  | X  (Control group – paper diaries) | X  (Online) |  |
| **Internet Evaluation and Utility Questionnaire** |  |  |  |  |  | X |  |  |  |
| **Internet Impact and Effectiveness Questionnaire, Internet Evaluation and Utility Questionnaire, and the Internet Intervention Adherence Questionnaire (Phase I and II)** |  |  |  |  |  |  |  | X |  |
| **Exit interview regarding experience with SHUTi program (Phase I only)** |  |  |  |  |  |  |  | X |  |
| **Dichotomous Yes/No Relapse Assessment** |  |  |  |  |  |  |  |  | X  (If YES, TLFB will be administered) |
| **TLFB** |  |  |  |  |  |  |  |  | X |

* Web program – SHUTi and Insomnia Education Only Program
